# Documenting Differences between Early Stone Age Flake Production Systems: An Experimental Model and Archaeological Verification

**DOI:** 10.1371/journal.pone.0130732

**Published:** 2015-06-25

**Authors:** Darya Presnyakova, Will Archer, David R. Braun, Wesley Flear

**Affiliations:** 1 Department of Early Prehistory and Quaternary Ecology, University of Tübingen, Schloss Hohentübingen, Tübingen, Germany; 2 Department of Human Evolution, Max Planck Institute for Evolutionary Anthropology, Leipzig, Germany; 3 Center for the Advanced Study of Hominid Paleobiology, George Washington University, Washington, DC, United States of America; 4 Department of Archaeology, University of Cape Town, Rondebosch, Western Cape, South Africa; Université de Poitiers, FRANCE

## Abstract

This study investigates morphological differences between flakes produced via “core and flake” technologies and those resulting from bifacial shaping strategies. We investigate systematic variation between two technological groups of flakes using experimentally produced assemblages, and then apply the experimental model to the Cutting 10 Mid -Pleistocene archaeological collection from Elandsfontein, South Africa. We argue that a specific set of independent variables—and their interactions—including external platform angle, platform depth, measures of thickness variance and flake curvature should distinguish between these two technological groups. The role of these variables in technological group separation was further investigated using the Generalized Linear Model as well as Linear Discriminant Analysis. The Discriminant model was used to classify archaeological flakes from the Cutting 10 locality in terms of their probability of association, within either experimentally developed technological group. The results indicate that the selected independent variables play a central role in separating core and flake from bifacial technologies. Thickness evenness and curvature had the greatest effect sizes in both the Generalized Linear and Discriminant models. Interestingly the interaction between thickness evenness and platform depth was significant and played an important role in influencing technological group membership. The identified interaction emphasizes the complexity in attempting to distinguish flake production strategies based on flake morphological attributes. The results of the discriminant function analysis demonstrate that the majority of flakes at the Cutting 10 locality were not associated with the production of the numerous Large Cutting Tools found at the site, which corresponds with previous suggestions regarding technological behaviors reflected in this assemblage.

## Introduction

Acheulean lithic technologies are first recognizable in East Africa by approximately 1.7 Ma at the sites of Kokiselei 4, West Turkana, Kenya [1] and Konso Gardula in Ethiopia [2]. The Acheulean persisted for more than a million years as evidenced by east African later Acheulean sites between 500–200 ka such as Olorgesailie Member 14 [3], Isimila [4], sites LH and FS in the Kapthurin formation [5] as well as Wonderwerk Cave [6] in South Africa and 8-B-11 in Sudan [7].

The production of Acheulean bifacial Large Cutting Tools [8] (hereafter “LCTs”) remains the most characteristic and recognizable technology by which Acheulean industries are traditionally identified [9]. However, innovations associated with the appearance of the Acheulean are manifested within a broader technological system than production of the bifacial forms themselves [10–12].

Relative to chronologically preceding industries, Acheulean technologies are argued to represent increased complexity in (1) raw material procurement and selection patterns [13–18], (2) provisioning systems and mobility [19,20] as well as (3) tentatively, a possible increase in levels of socially mediated information transfer within and between artifact producing groups [21–23].

The Acheulean is also associated with the emergence of broadly systematic bifacial shaping. In the context of handaxe production this shaping initiates through the roughing out of selected blanks, and is followed by a sequence of finishing stages aimed at thinning, as well as refining bilateral and bifacial symmetry [24]. It has been suggested that *debitage* is the intentional fracturing of a volume of raw material to produce flakes, whereas *shaping* is applied to reduce a volume of raw material with a template or notion of preconceived bifacial form [24].

Cores, viewed independently, are capable of revealing information about morphology associated with reduction strategy, intensity and perhaps even function in the context of certain core tools [25–28]. However, a broader understanding of Middle Pleistocene technological organization is attainable only by studying all parts of the technological system[29].

In this vein the byproducts of bifacial shaping that are unequivocally associated with LCT production x and their distribution on the landscape—are as important proxies for Acheulean technological behavior as the shaped LCTs themselves [30,31]. Conversely, detached pieces within Acheulean assemblages that are not byproducts of LCT production contain valuable information about variability in Acheulean technological behaviors [13,32].

Here we develop an experimental framework using morphometric data and multivariate statistics to quantify variation in flakes produced (1) within the application of roughing out and bifacial shaping strategies as well as (2) via a discrete set of flake production strategies identified within Oldowan industries. To test the validity of our methodological framework as well as its suitability to archaeological materials, we analyze the archaeological collection of whole flakes from the well-studied Mid-Pleistocene site of Cutting 10 from Elandsfontein [18,33].

Previous descriptive and typological analyses of the materials from Cutting 10 suggested that the numerous bifacial tools recovered at this locality were not produced there [33]. The Elandsfontein locality is a well-studied collection of Mid-Pleistocene tools that have been recovered in association with an extensive fossil assemblage [34,35]. Our analysis supports previous assertions that LCT production occurred elsewhere on the landscape, and that LCTs were transported to the Cutting 10 locality in finished form prior to discard [33]. Additionally, flake products were produced on site that had no technological association with the LCT component of the Cutting 10 assemblage. We describe the application of our methodology to the Cutting 10 collection to demonstrate its utility to documenting landscape scale variation in patterns of Middle-Pleistocene tool manufacture, maintenance and discard in ways that are quantitatively replicable and comparable.

Importantly, in this paper the ‘Modes’ terminology will be used as a reference to technological forms. Graham Clark defined Oldowan or “core and flake” technology as Mode 1, and bifacial industries as Mode 2 [36,37]. Importantly, the “Mode” terminology used in this paper is not applied in the sense of a evolutionary trajectory [38], or to associate industries with distinct hominin groups [39]. Here we use the terms Mode 1 and Mode 2 for heuristic purposes only as baseline descriptive concepts [40]. Further, instead of referring to bifaces we will use the term “LCT” [10].

## Background

In the 1950s-1970s Acheulean sites were traditionally and widely identified by the presence/absence of characteristic bifacial forms [41].Characteristic LCTs were subsequently divided into categories ranging from primitive to more advanced [42,43]. For example Kleindienst used LCT percentages to classify sites at Olduvai Gorge as either Acheulean or Oldowan [44]. Mary Leakey used the presence/absence criteria in association with LCT frequencies to define the term Developed Oldowan [43]. According to Leakey “Developed Oldowan A” was an Oldowan toolkit that also had proto-bifacial elements present. “Developed Oldowan B” necessarily had to have a certain percentage of bifacial pieces within the toolkit More recently researchers have suggested that “Developed Oldowan” in general should rather be referred to as Acheulean [45].

The technological approach that flourished during the 1980–90s focused on LCT variability purportedly associated with differences in tool function [46–50], factors driving morphological variability [14,15,51–57] and patterns of reduction [11,12,45,58–61]. However, we outline two scenarios below that potentially warrant formulation of a different approach to investigating Acheulean technological behavior.

### A. Acheulean sites where diagnostic LCTs are absent

Numerous sites have been classified as Acheulean, but their attributions remain contentious due to lack of traditional characteristics, namely, absence of classical LCTs. For example, as LCTs are often made on flake blanks it was suggested that the presence of systematic large flake production may be a diagnostic criteria of the Acheulean technocomplex in contexts where the LCTs themselves were not present [62,63]. A number of Pleistocene sites have consequently been identified as being representative of the Acheulean solely on evidence for large flake production. Some examples include FC West, Olduvai Gorge [45], Kokiselei 4, West Turkana [64], and FxJj 63 in Koobi Fora [62].

Examples of Acheulean sites with no LCTs are also known from much younger Middle Pleistocene contexts. For instance, The Nadung’a 4 locality in West Turkana is a Middle Pleistocene site dated to around 700 ka. Artifact assemblages at Nadung’a 4 contain denticulates and notches, but no LCTs have been identified [65]. Likewise, layers V-5 and V-6 at Gesher Benot Ya’aqov, Israel contain no LCTs but were assigned to the Acheulean through characterization of technological aspects of flake collections [63]. The purported absence of LCTs at many Middle Pleistocene sites in eastern Asia is significant [66], as well as the presence of relatively crudely shaped LCTs at some of the Korean sites, such as Imjin/Hantan River Basin [67].

A plausible explanation for the lack of characteristic LCTs within the assemblages from these localities is that only the early stages within the LCT *chaîne opératoire* are present there. These assemblages may represent windows on the LCT production sequence where blanks or preforms had not yet taken on recognizable bifacial characteristics. By ‘blanks’ here we mean large flakes or split cobbles, and by ‘preforms’ we refer to pieces that have been coarsely shaped or ‘roughed out’ [24].

It has also been suggested that the onset of LCT production represents a shift in hominin raw-material transport decisions [16,17]. It is therefore both plausible and probable that sites exist where both LCTs and/or the blanks intended for LCT manufacture had been transported away from a locality where bifacial strategies were nevertheless practiced [63]. In these contexts, the residual elements of LCT *chaîne opératoire* would be the detached pieces associated with the shaping of LCTs [31,32]. The relationships between flake characteristics and specific reduction processes have been investigated by several authors in the study of more recent bifacial assemblages [68–70]. Therefore, we extend these inferential links here by widening the spectrum of assemblage characteristics through which *shaping* can accurately be identified in an Acheulean context.

### B. Acheulean sites where bifacial shaping products co-occur with core and flake technologies

Distinguishing the relative proportions of flakes associated broadly with bifacial shaping from those associated with ‘core and flake’ debitage strategies may seem qualitatively trivial [71,72]. However, no quantitative method currently exists to quantify these distinctions.

It has been proposed that the emergence of bifacial technology coincided with the demise of core and flake technology [38,73]. This proposed linear trajectory of technological evolution is challenged by Pleistocene sites where simple core and flake products are present in association with LCT manufacture. Examples include Olorgesailie, Kenya [20], Peninj, Tanzania [58], Koobi Fora, Kenya [74] and Gadeb, Ethiopia [59]. In the Middle Awash, there are also Early and Middle Pleistocene sites where Oldowan core and flake technologies overlap chronologically with bifacial technologies [75]. For instance the site of Bodo (> 600k) exhibits only simple core and flake technologies [76]. An important characteristic these examples share is that multiple technological strategies were practiced at the same site. The co-occurrence of several technologies at certain Middle-Pleistocene sites may have been related to the co-existence of multiple hominin lineages [77], the influence of variable environmental settings [78–80] or some other uninvestigated phenomenon.

## Materials

### Experimental assemblage

We used an experimental assemblage of Mode 1 and 2 flakes to model the effects of sets of independent measures of flake shape on technological group separation. Importantly, within the experimental assemblage the technological group affiliation of each individual flake is known.

The experimental assemblage of Mode 1 and Mode 2 flakes was produced by two experienced knappers (both with more than 10 years of knapping experience). While knapping, the two knappers had no knowledge of the intended usage, and specific archaeological application of the experimental assemblages they were generating. They therefore did not produce flakes in accordance with a specific morphological template. To make the experimental flakes, we used quartzite and silcrete, two of the raw materials exploited at Elandsfontein Cutting 10. In producing the Mode 2 experimental assemblage, the knappers followed the LCT production sequence previously documented for Elandsfontein Cutting 10 by Archer and Braun [18]. The Mode 2 assemblage included 98 flakes generated within the production of 28 LCTs. LCTs were produced by first using a hard hammer and then switching to soft hammer on large (>10cm) side-struck flakes (flakes where the technological length is perpendicular or close to being perpendicular to the maximum length). Side-struck flakes are the dominant blank-form used within the Elandsfontein Cutting 10 LCT collection [18]. Importantly, Mode 2 flakes resulting from roughing out (also referred to as *ébauche)* and thinning (also referred to as *façonnage*) [81] were combined for analytical purposes. Thus, morphological variability within the Mode 2 sample was maximized, to incorporate the maximum potential overlap with Mode 1 flakes in the discrimnant model. In this way shape variation in Mode 2 flakes related to the activities of roughing out and subsequent shaping were both included.

Mode 1 flakes (n = 149) were produced from the reduction of 12 (< 8cm) riverine cobbles. Rounded cortex on many of the cores and flakes from the Cutting 10 assemblage indicate that riverine cobbles were the initial form of many of the cores. To account for some of the documented variation in Mode 1 core reduction strategies [45,82–84] several techniques were utilized including unifacial unidirectional, multidirectional and centripetal (discoidal) [45]. Some of these reduction strategies such as discoidal and multidirectional variants have been identified within the Elandsfontein Cutting 10 collection.

The experimental assemblage consisted of whole flakes. Flakes were considered ‘whole’ if all of the relevant variables could be measured. The whole flakes were measured using a variety of caliper and digital imaging techniques (i.e. measurements that were made using the photos of flakes). In addition, whole flakes smaller than 2 cm were excluded as it has been suggested that caliper measurements are less reliable and more subjectively variable on flakes smaller than 2 cm [85]. In addition, the digital measurements of curvature used in this study were substantially less precise on small flakes (Table 1).

**Table 1:** Descriptive parameters of the experimental assemblage.

Experimental flakes:	silcrete(n = 77)		quartzite (n = 72)	
Mode 1 (n = 149)	Mean	Sd	Mean	Sd
Length*	57.38	21.74	62.01	19.92
Width	48.95	21.39	59.85	23.45
Thickness	13.98	6.17	16.5	6.43
Mass	66.15	78.91	97.69	88.07
Experimental flakes:	silcrete(n = 93)		quartzite (n = 5)	
Mode 2 (n = 98)	Mean	Sd	Mean	Sd
Length*	36.24	15.08	44.11	13.58
Width	26.77	10.04	32.55	6.42
Thickness	5.38	2.78	7.7	2.98
Mass	8.78	9.35	8.3	4.49

*Technological length, not maximum length.

### Archaeological collection

**Fig 1:**
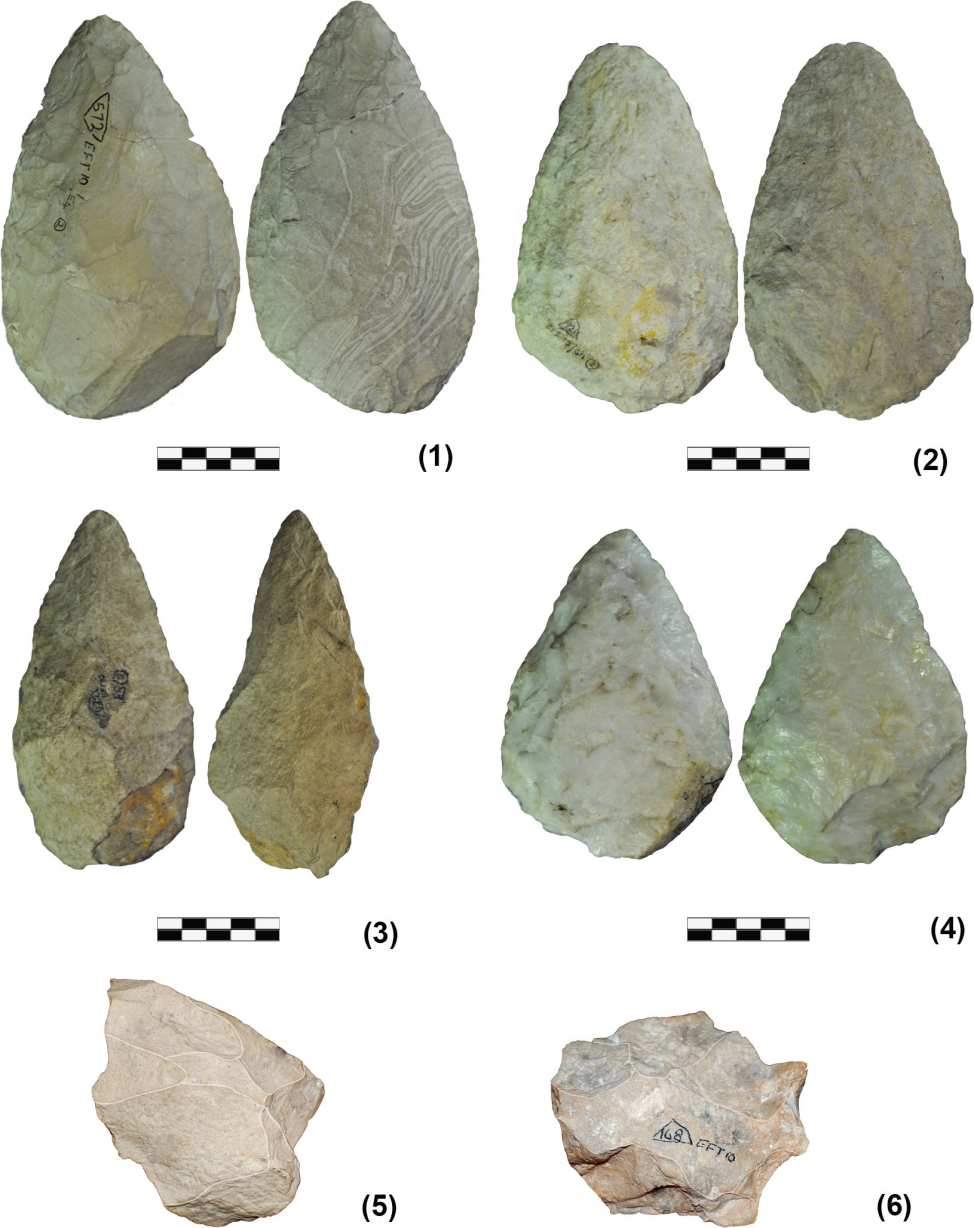
Archaeological specimens. The figure displays Mode 1 cores and Mode 2 LCTs from Elandsfontein Cutting 10 assemblage: (1) EFT_572 (Mode 2:silcrete), (2) EFT_921 (Mode 2:silcrete), (3) EFT_622 (Mode 2:silcrete), (4) EFT_579 (Mode 2:quartz), (5) EFT_182c (Mode1:siclrete), (6) EFT_168a (Mode 1:silcrete).

Elandsfontein is a Mid-Pleistocene dunefield on the West Coast of South Africa that has a bio-stratigraphic age determination between 600kya and 1mya [35]. There is a variety of raw materials represented in the stone tool assemblage. At Elandsfontein Cutting 10 the most frequently used materials were a sub-volcanic rock referred to as quartz porphyry, silcrete, quartzite, quartz and substantially smaller amounts of hornfels (S1 Table). The Elandsfontein Cutting 10 lithic assemblage is from an excavation of *in situ* deposits in the south-east portion of the dune field [34]. The archaeological assemblage from Cutting 10 (Fig 1) consists of 208 artifacts, 66 of which are LCTs, 29 cores and 129 flakes and flake fragments [18,34]. The cores can be classified broadly as discoidal and multidirectional, with short flaking series that do not exceed 3–5 removals. However, multiple series may appear on a given core. Of the 129 flakes and flake fragments, the majority have either post depositional breakages or fractures that potentially occurred during knapping or some other activity (e.g. *siret* flakes). In order to measure all relevant attributes our analysis included the 34 complete archaeological flakes from the Elandsfontein Cutting 10 collection (Table 2). All analyzed artifacts are currently stored at the Department of Archaeology, University of Cape Town (Cape Town, South Africa). Access to the materials was gained through an agreement between the authors and the Department of Archaeology, University of Cape Town. Permitting details associated with the 1966 excavation of Ronald Singer and John Wymer, are no longer available at the Iziko Museum, Cape Town.

**Table 2:** Descriptive parameters of the archaeological assemblage.

Archaeological flakes(n = 34)	All raw mat.		quartzite		silcrete		quartz^1^	
	Mean	Sd	Mean	Sd	Mean	Sd	Mean	Sd
Length*	46	15.62	44.05	20.56	46.3	15.7	38.06	10.83
Width	39.68	10.61	59.73	19.95	38.17	9.19	37.67	3.74
Thickness	13.27	4.89	23.25	5.45	12.6	4.34	10.9	2.81
Mass	34.95	25.25	82.75	64.7	32.48	21.07	23.85	5.59

*Technological length, not maximal length.

^1^Hornfels, quartz porphyry and sandstone were only included into “all raw material” category as sample size representing each of these raw materials equals to 1.

The Elandsfontein Cutting 10 assemblage is particularly suited to investigations into variation in Mid-Pleistocene core reduction strategies for three principal reasons. (1) The collection is comprised of a variety of raw-materials; (2) Sources of stone suitable for artifact manufacture are unavailable within 10 km of the locality [33,86], and (3) both primary and secondary sources of raw material were exploited resulting in marked variability in the range of blank forms exploited [18].

## Methodology

### Variable selection

Mode 1 cores in general encompass a large variety of core reduction techniques including unifacial, multidirectional and bifacial partial amongst many other variants [45,82,87–89]. However, since these strategies were not applied to achieve a discrete form [24], there are no established guidelines whereby Mode 1 flakes can be identified through their morphological characteristics alone. In contrast, Mode 2 LCT production is applied to develop bifacial and bilateral symmetry of an LCT, while maintaining surface convexities on the LCT [24]. These dual objectives require removing flakes during LCT production, which are characterized by a modal shape or set of shapes.

**Fig 2:**
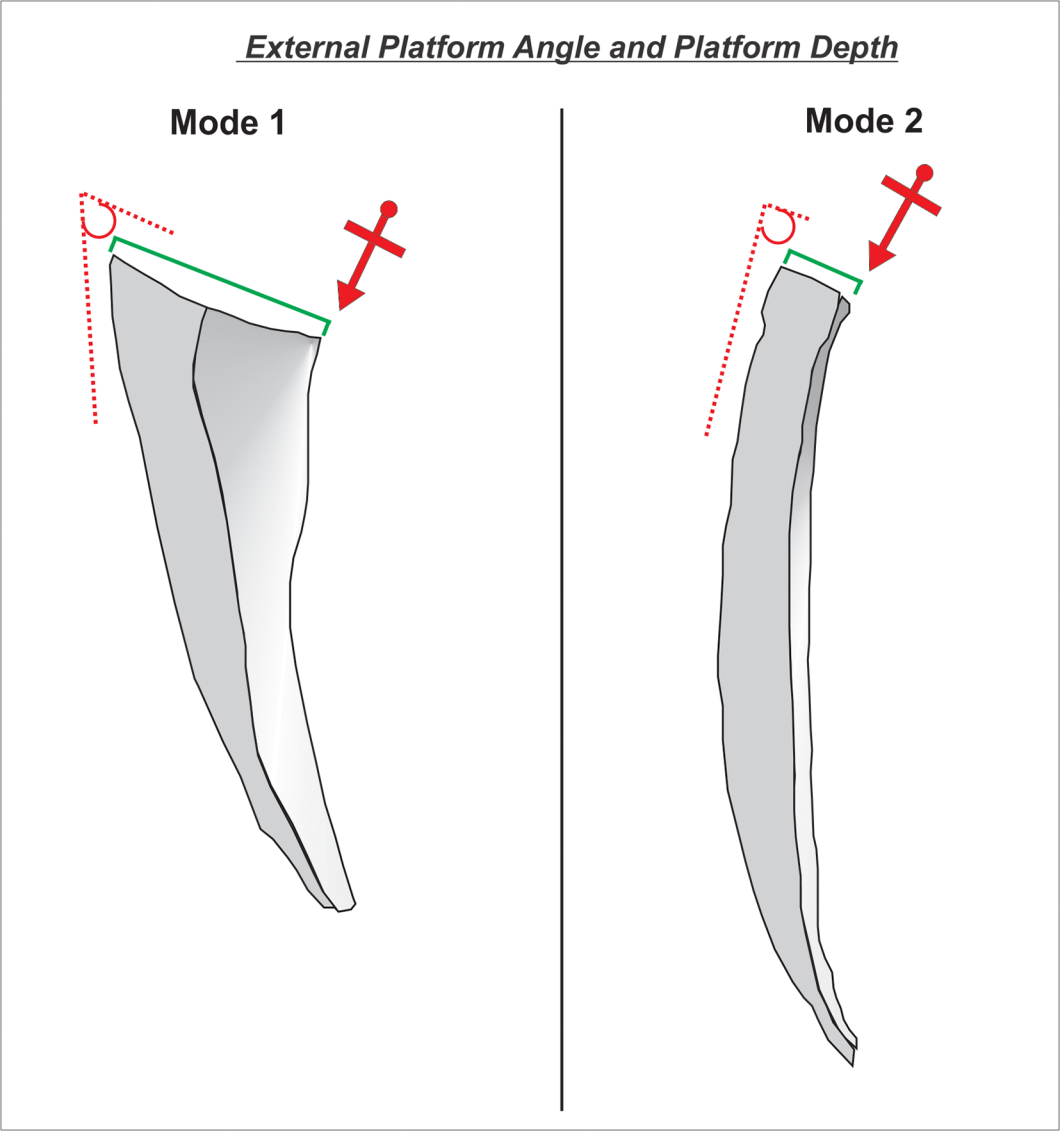
External platform angle and platform depth. Dashed line represents EPA, solid line represents platform depth and arrows are points of percussion.

**Fig 3:**
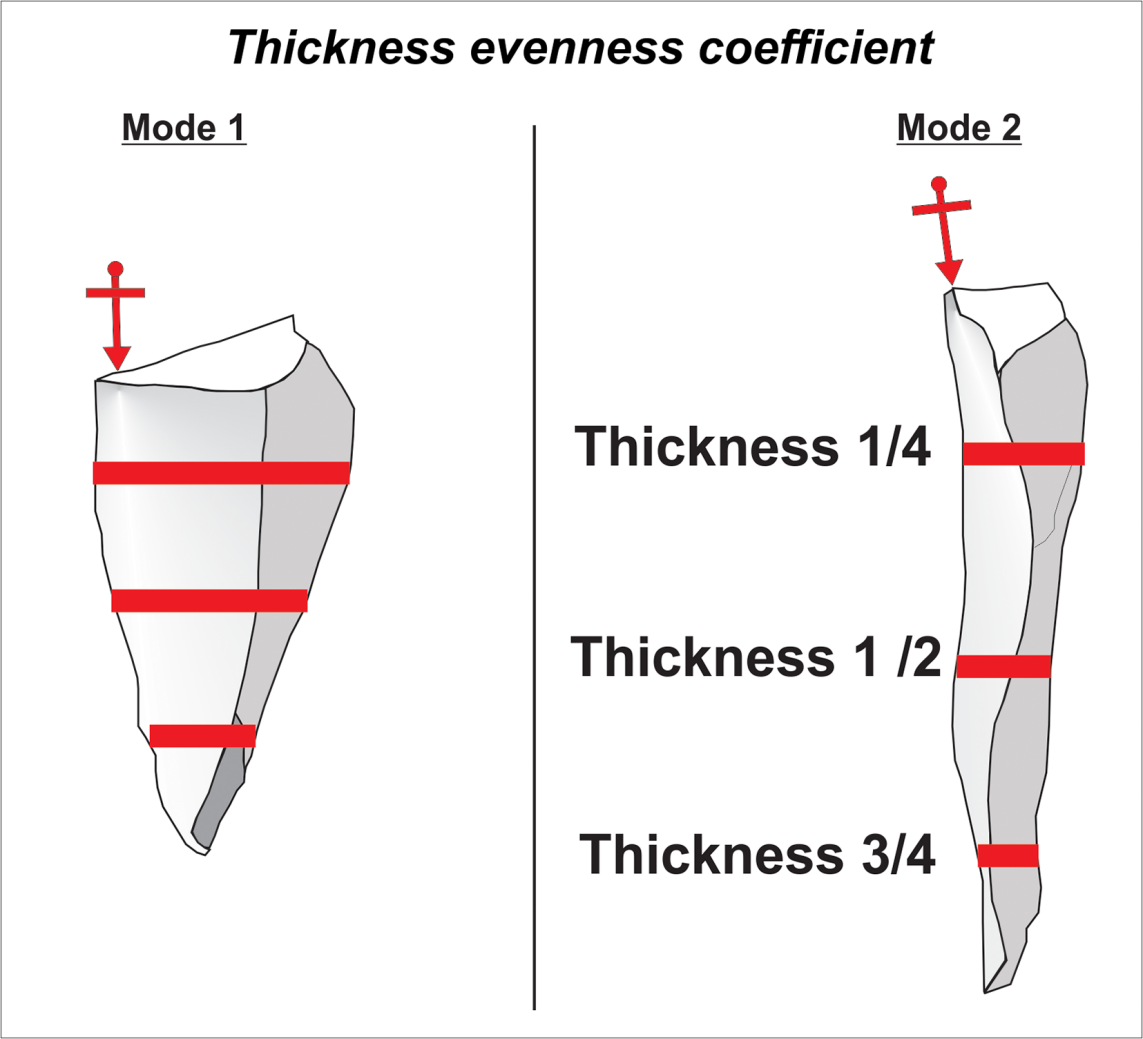
Thickness evenness coefficient. Arrows show points of percussion, whereas solid lines represent places on a flake where thickness was measured.

**Fig 4:**
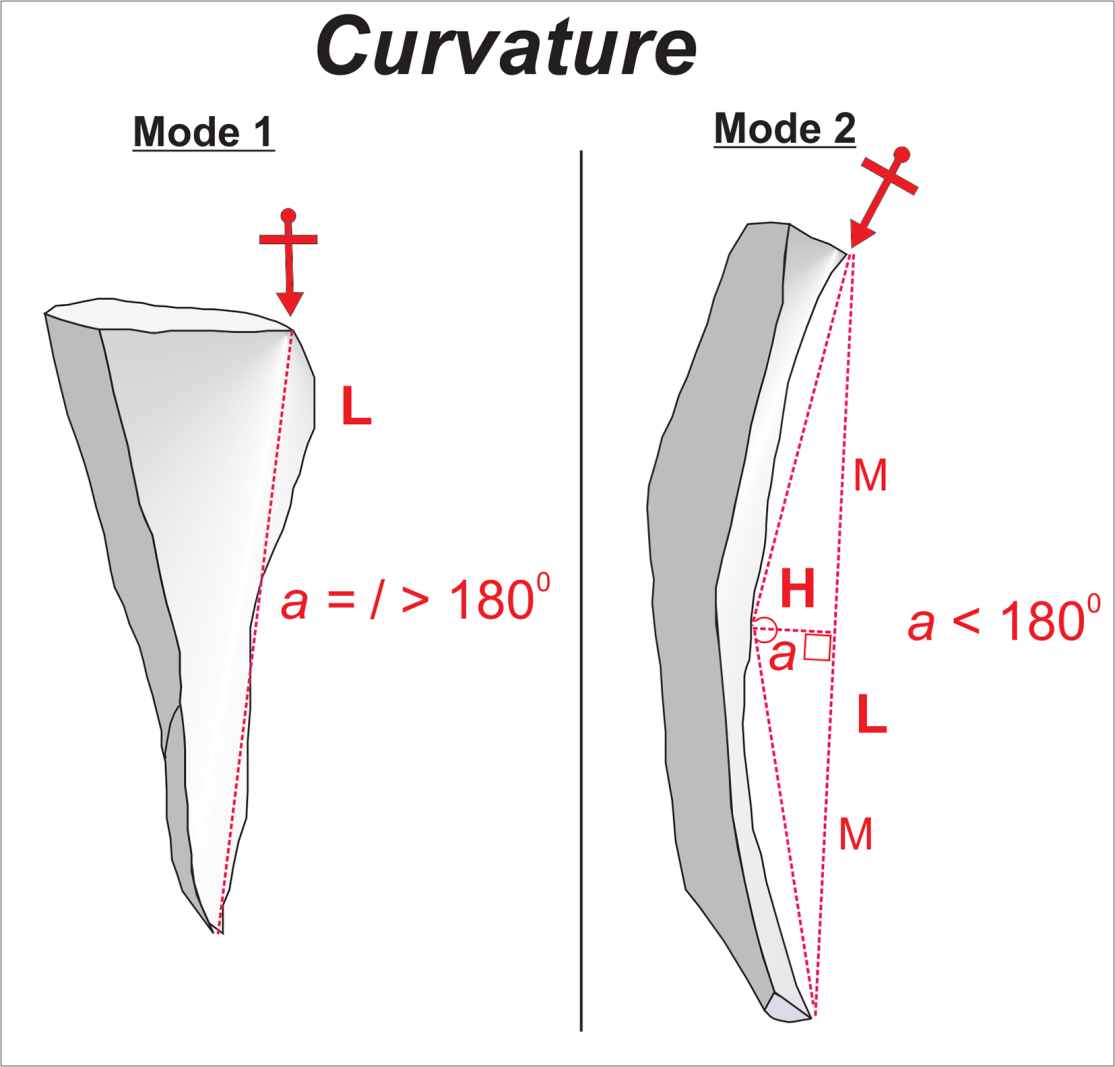
Curvature. Arrows represent points of percussion, dashed lines are the outlines of right triangles composed from the technological length and the height measurements.

In terms of modal shape, Mode 2 whole flakes tend to be long and wide, with a relatively small mass, small platforms, concave profiles and are thin relative to Mode 1 flakes [58,90]. However, it would not be possible to incorporate *all* these variables into multivariate analysis as (a) they are correlated with one another, which would result in collinearity, and (b) variables such as length, width or mass are not reliable predictors of shape in themselves as the aspects of shape that they influence are often correlated with overall flake size (e.g. allometry). As such, we have selected a series of variables that focus specifically on the technologically relevant components of whole flake shape:

1. External platform angle (hereafter “EPA”) (Fig 2): EPA is the angle measured between the striking platform surface and dorsal surface of a flake directly behind the point of percussion [91–97]. It is one of the few variables which is directly controlled by the knapper [91]. Experimental research has demonstrated that EPA influences both flake size and flake morphology [92,93,95].
2. Platform depth (Fig 2): We measured platform depth from the point of percussion to the point of intersection between the platform surface and the dorsal surface of the flake [91]. Experiments have demonstrated that platform depth correlates with overall flake size i.e. an increase in platform depth correlates with an increase in flake mass [91,93]. Prior to the analyses presented here, platform depth was transformed to minimize the effects of allometry (hereafter when we discuss platform depth it is always size adjusted). The variable was standardized by dividing it by the geometric mean of all size related variables such as length, width, thickness and platform width [98]. This size adjustment enables the documentation of shape differences associated with platform variability that are not directly related to size [99].
3. Interaction between platform depth and EPA: It has been suggested that flakes with large EPAs generally are large in size [91]. However, depending on the magnitude of platform depth flakes with large EPAs will be either thick with pronounced bulbs of percussion (observed at large values of platform depth) or will have a large surface areas (observed at small values of platform depth) [91,94]. In the course of bifacial reduction the bi-convex shape of the core–with necessarily acute edges–does not usually allow for large EPAs (>80°). This means that by default Mode 1 flakes will generally have more varied and larger EPAs than Mode 2. Importantly though, in comparison to Mode 1, the EPA values from Mode 2 flakes tend to be large relative to platform depth. In terms of multivariate statistics, the presence of a potentially significant interaction does *not* imply a correlation of two variables [100], it suggests that the effect of one influential variable on a response is not the same for all respective values of another influential variable. The interaction between platform depth and EPA was therefore included in the models formulated in this study.
4. Thickness evenness coefficient (Fig 3): This variable captures variation in flake thickness along the technological length axis. Thickness was measured at 25%, 50%, and 75% of the technological length. The standard deviation was then calculated for these three values. Low values indicate that thickness measurements do not vary greatly along the technological axis whereas high values indicate substantial variation in thickness along the technological axis. High values may be explained by flakes where the volume is concentrated at one point in the flake (e.g. bulb of percussion). Maintenance of the bi-convex section of an LCT requires removals with a relatively evenly distributed thickness, thus Mode 2 flakes are more likely to have lower values. Eren and Lycett [101] documented similar patterns of thickness variation within an individual flake in other technologies where flake shape is maintained by the knapper [101].
5. Interaction between platform depth and thickness evenness: The predictive capacity of one variable–may be augmented at either high or low values of the other variable. Therefore, the interaction between the two variables was included.
6. Curvature (Fig 4): The formula for calculating curvature on flakes was introduced by Andrefsky [102,103]; and here calculated on images of flake profiles using Image J 1.43u software (S1 Text). The curvature variable is based on predictions regarding how the morphology of a core surface is maintained. Core surface maintenance contingently affects the shape of the flakes removed from it [95,104,105]. For example during *façonnage*, removals frequently invade beyond the midline of the plano-convex surface of an LCT [24]. Consequently, flakes associated with shaping tend to have curved profiles [63,102]. Since Mode 1 flakes are not associated with shaping of a form, they typically have either flatter profiles or convex ones due to the often pronounced character of the bulb.

### Discriminant Function Analysis

Discriminant function analysis is a multivariate statistical technique that constructs linear functions which maximize separation between pre-existing groups [106,107]. The central principle of the discriminant function analysis is that linear combinations of the predictor variables are constructed in a way that between group variance is maximized [108]. Once the discriminant function is developed, using cases of known group classification, the classification of individual observations results in a probability of group affiliation [106]. A key utility of discriminant function analysis is that it enables classification of data that was not used to build the discriminant model. In this study, the model was built using the previously described experimental data set. This was followed by the classification of an out-group of experimental flakes to determine the number of flakes classified correctly. This cross validation methods serves to verify the discriminant function. Finally, archaeological flakes are classified using the same variables and then the discriminant function analysis produces a value that estimates the likelihood of group association.

The five predictor variables used in the development of the discriminant function analysis were EPA, platform depth (standardized by the geometric means of several size dependent variables), curvature, and thickness evenness (hereafter “test predictors”). Test predictors are included in the model as there is a specific question or hypothesis about them outlined in a study. On the contrary, the control predictors are not related to a specific hypothesis and are only considered in statistical models to monitor their potential effect [109]. Since we used quartzite and silcrete to create our experimental assemblage, in the model we included raw material as a control predictor. In this model, group membership (Mode 1 or Mode 2) of the flakes was the response. The number of whole flakes included in the test was 247.

As it has been already mentioned, platform depth was standardized with the geometric mean. The distributions of all predictor variables were checked for outliers, homogeneity of variances and the assumption of normality [110]. Consequently, platform depth and thickness evenness were transformed with a square root transformation to achieve distributions that approached the assumption of a normal distribution. To homogenize the predictors of different scales all covariates used in the model were standardized with the z-transformation which makes the covariate’s mean equal 0 and standard deviation equal 1 [100]. Discriminant function analysis was performed R programing interface [111] using functions available in the “Mass” package, namely, ‘lda’ and ‘lda predict’ [112].

### Generalized Linear Model

In addition to the discriminant function analysis we analyzed the data using the Generalized Linear Model with a binomial error structure and the logit link function [113]. We chose to use logistic regression as this enabled us to interrogate *how* the predictors (e.g. thickness evenness) operated and interacted with one another in distinguishing technological groups. The Generalized Linear Model was used to investigate how the predictors of curvature, platform depth, thickness evenness and EPA influenced group membership of Mode 1 and 2 flakes. Raw material was again added as a control predictor.

In our experimental assemblage there was a predominant use of flake blanks that retain only dorsal cortex (or remnants thereof) for LCT production. Mode 1 flakes were produced on cobbles as well as natural outcrops. Due to this necessary difference in blank forms used for Mode 1 and Mode 2 experimental assemblages, there was an uneven distribution of cortical and non-cortical flakes between the two groups. Cortex presence therefore potentially influences group membership as a response. In other words, Mode 1 flakes on aggregate tend to retain more cortex simply because there was more cortex present on the initial Mode 1 blanks used. Thus, we included cortex presence as another control predictor.

To verify the significance of the full model (the model where all predictors are included) we used a likelihood ratio test, comparing its deviance with that of the null model, which comprised of the intercept only. We tested for several interactions including (a) platform depth with EPA as well as (b) platform depth with thickness evenness. To test for the significance of these interactions we compared the full model's deviance with that of a corresponding reduced model comprising no interactions.

Overall, in the model (both the full model and the reduced model), we again used the platform depth variable standardized by the geometric mean to avoid size related effects. Similarly, to the discriminant function analysis, all covariates were again standardized with the z-transformation. All Generalized Linear Model variations derived in this study were checked for a series of assumptions regarding data distribution and model stability including collinearity, DfBetas, leverage and overdispersion [110,114–116]. The model was fitted in R [111], using the “glm” function.

## Results

### Discriminant Function Analysis

**Fig 5:**
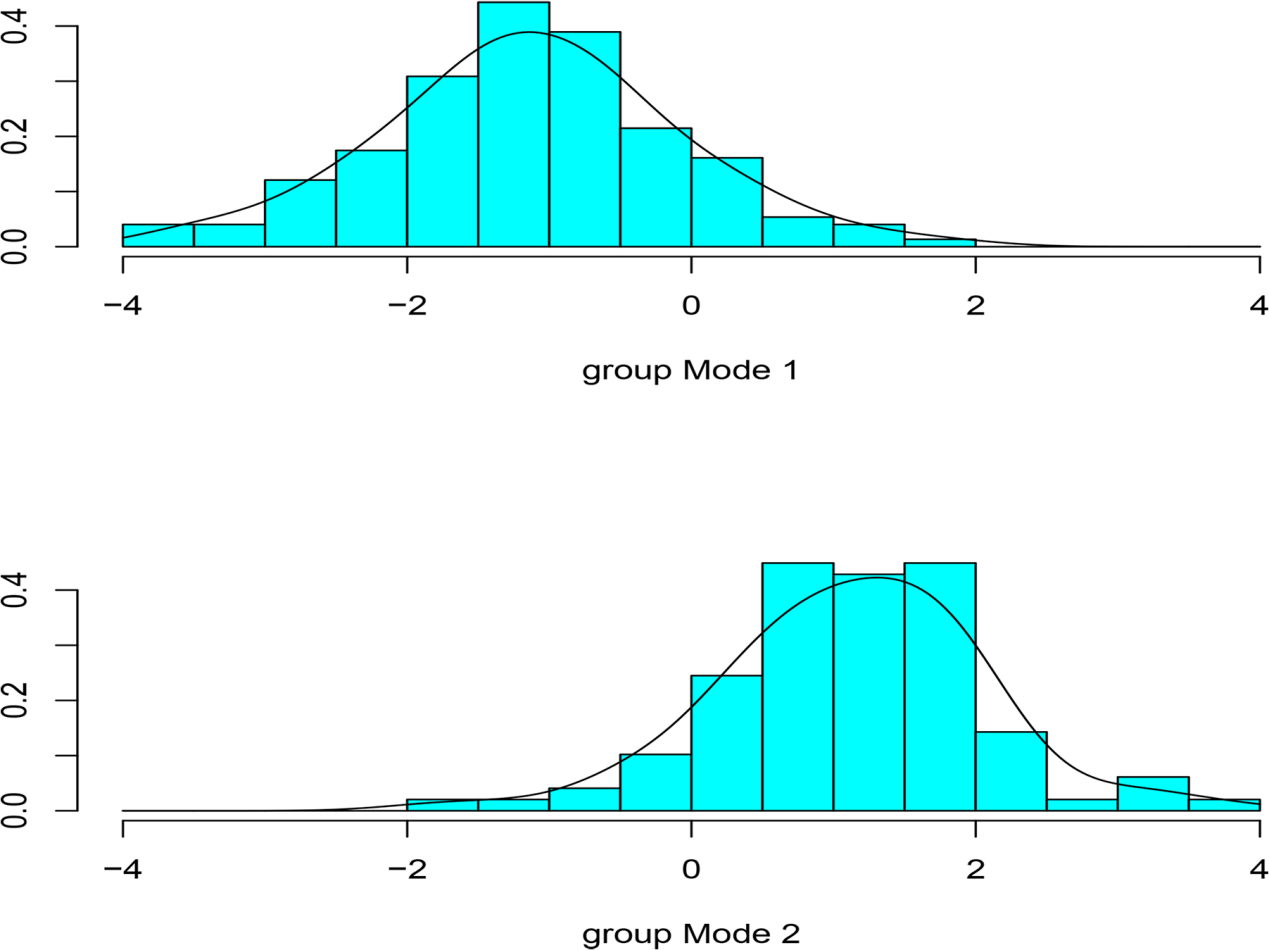
Discriminant function analysis-Experimental. A histogram showing the results of the discriminant function scores with the inclusion of experimental data only.

The discriminant function analysis resulted in an 87.4% success rate in experimental flake classification (Fig 5). A subsequent analysis with jackknife re-sampling, a cross validation method of leaving one observation out [108], resulted in a similar percentage of correctly classified cases. The jackknife method prevents a situation where the model is tested against the same specimens that are used to create the model.

As raw material type was identified as a significant predictor (Table 3), we considered the percentage of correctly classified cases using a subset of the data that included only silcrete flakes (therefore raw material variability was removed as a predictor). In that data subset the percentage of correctly classified classes is 84% and when using jackknife re-sampling 82% of specimens were correctly classified. Therefore, with and without the inclusion of raw material variation, the standardized coefficients of the linear discriminants show extremely similar results. In the dataset where the raw material is not included the effect sizes of the individual predictors is the following: the curvature variable plays the most prominent role in separating the technological groups followed by thickness evenness, EPA and platform depth (Table 4).

**Table 3:** The results of the discriminant function analysis on experimental assemblage showing the loadings of the linear discriminant 1.

	LD
Raw material	1.02
Curvature	-0.78
Thickness even.	-0.51
EPA	0.33
Platform depth	-0.19

**Table 4:** The data subset showing the loadings of the liner discriminant 1 for only those experimental flakes made on silcrete.

	LD
Curvature	-0.79
Thickness even.	-0.72
EPA	0.24
Platform depth	-0.21

### Generalized Linear Model

**Fig 6:**
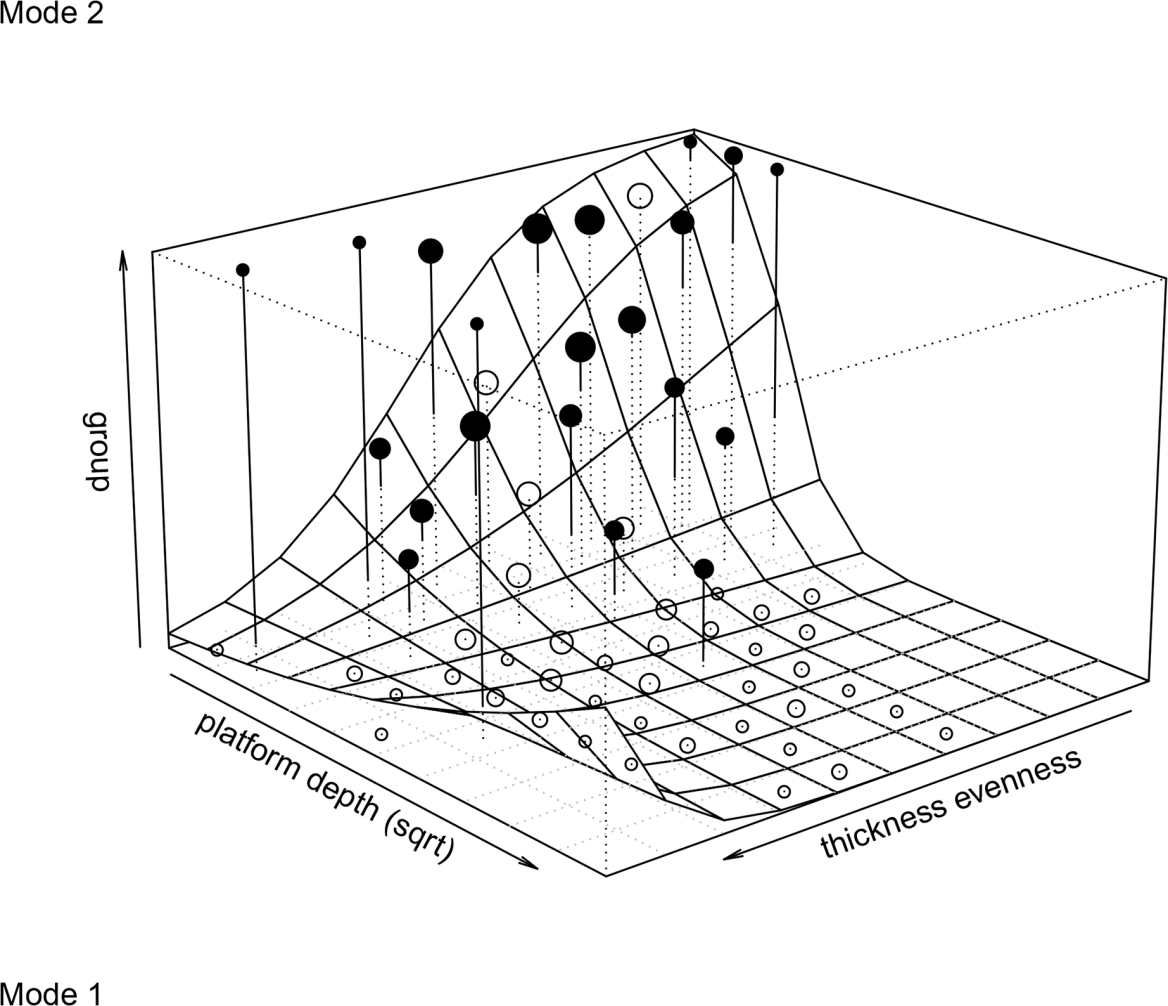
Generalized Linear Model-Platform depth * thickness evenness. The plot represents the effect of platform depth and thickness evenness on group separation at the average of EPA, curvature and raw materials. Points depicting the average response per cell of the fitted surface can be scaled according to the number of data in the respective cell and are depicted as filled when the average is above the fitted model and as open points when they are below. Points are connected to the bottom of the figure by a line, which is dashed between the bottom of the figure and the fitted surface, and solid above it.

A likelihood ratio test comparing the null and the full Generalized Linear Model was highly significant (χ^2^ = 225, df = 7, P< 0.001) (Table 5). The reduced model that did not include interactions revealed that curvature, thickness evenness, platform depth and EPA were all significant predictors (see Table 6 for coefficients). There was a significant interaction between thickness evenness and platform depth. The likelihood ratio test comparing the reduced (no interaction) and full models was also highly significant (χ ^2^ = 239, df = 1, P< 0.01). The significance of the interaction implies that at greater platform depths, thickness evenness has a greater impact on the separation of whole flakes in Mode 1 and Mode 2 groups (Fig 6). As raw material was a significant predictor, we tested the model holding raw-material constant. Table 7 shows the result of the data subset that includes only the silcrete assemblage. The effect sizes and significance of the individual predictors is similar.

**Table 5:** The results of the Generalized Linear Model (group~ plat.depth*thick. eve. +epa + curvature+ cortex+ raw material) on experimental assemblage.

Effect ^(2)^	Estimate	SE	t	P	lower CI	upper CI
Intercept	-1.264	0.351	^(1)^	^(1)^	-2.019	-0.624
platform depth ^(3)^ ^,^ ^(4)^	-0.330	0.288	^(1)^	^(1)^	-0.911	0.229
thickness evenness	-1.919	0.436	^(1)^	^(1)^	-2.895	-1.149
Curvature	-1.955	0.396	-4.934	0.000	-2.804	-1.237
Epa	-0.895	0.358	-2.502	0.012	-1.655	-0.236
Cortex	-2.053	0.877	-2.339	0.019	-3.865	-0.410
raw material	2.378	0.685	3.472	0.001	1.117	3.830
plat. dp:thick. eve	1.068	0.398	2.682	0.007	0.307	1.907

(1) not shown because of not having any meaningful interpretation

(2) all covariates were z-transformed to a mean = 0 and sd = 1, original means (sd) were: curvature: 175.67(10.61); epa: 68.46(14.90); thickness evenness: 2.71 (2.18); platform depth: 10.53(6.82)

(3) square root transformed prior to z-transformation

(4) standardized by the geomean

**Table 6:** The results of the reduced Generalized Linear Model, with no interactions.

Effect ^(2)^	Estimate	SE	t	P	lower CI	upper CI
Intercept	-1.097	0.330	^(1)^	^(1)^	-1.791	-0.488
platform depth ^(3)^ ^,^ ^(4)^	-0.691	0.250	-2.760	0.006	-1.214	-0.218
thickness evenness	-1.837	0.415	-4.422	0.000	-2.724	-1.078
Curvature	-1.697	0.350	-4.855	0.000	-2.441	-1.057
Epa	-0.885	0.329	-2.689	0.007	-1.580	-0.277
Cortex	-1.661	0.799	-2.080	0.038	-3.311	-0.159
raw material	2.121	0.637	3.330	0.001	0.938	3.466

(1) not shown because of not having any meaningful interpretation

(2) all covariates were z-transformed to a mean = 0 and sd = 1, original means (sd) were: curvature: 175.67(10.61); epa: 68.46(14.90); thickness evenness: 2.71 (2.18); platform depth: 10.53(6.82)

(3) square root transformed prior to z-transformation

(4) standardized by the geometric mean

**Table 7:** The results of the Generalized Linear Model including only silcrete flakes (n = 170).

Effect^(2)^	Estimate	SE	t	P	lower CI	upper CI
Intercept	0.357	0.363	^(1)^	^(1)^	-0.371	1.076
platform depth ^(3)^ ^,^ ^(4)^	-0.418	0.299	^(1)^	^(1)^	-1.035	0.152
thickness evenness	-2.049	0.488	^(1)^	^(1)^	-3.174	-1.211
Curvature	-2.046	0.456	-4.4853	0.000	-3.040	-1.231
Epa	-1.167	0.425	-2.7472	0.006	-2.093	-0.401
Cortex	-2.328	1.018	-2.2869	0.022	-4.429	-0.388
plat. depth:thick. eve	1.144	0.429	2.66522	0.008	0.348	2.072

(1) not shown because of not having any meaningful interpretation

(2) all covariates were z-transformed to a mean = 0 and sd = 1, original means (sd) were: curvature: 173.69(10.99); epa: 65.23(15.35); thickness evenness: 2.42 (2.07); platform depth: 8.44(5.57)

(3) square root transformed prior to z-transformation

(4) standardized by the geometric mean

The percentage of dorsal cortex was also a significant predictor. To isolate the effect of cortex as a predictor, we formulated an additional model that incorporated only non-cortical flakes from the experimental assemblage. Table 8 shows the data subset that includes only non-cortical flakes. In this model, our test variables (platform depth, thickness evenness, curvature and EPA) show similar patterns to the model that included both cortical and non-cortical flakes.

**Table 8:** Table shows the results of the Generalized Linear Model including only non-cortical flakes (n = 198).

Effect^(2)^	Estimate	SE	t	P	lower CI	upper CI
Intercept	-2.938	0.778	^(1)^	^(1)^	-4.619	-1.538
platform depth ^(3)^ ^,^ ^(4)^	-0.247	0.322	^(1)^	^(1)^	-0.899	0.371
thickness evenness	-2.585	0.638	^(1)^	^(1)^	-4.055	-1.531
Curvature	-2.198	0.480	-4.577	0.000	-3.251	-1.347
Epa	-1.126	0.424	-2.656	0.008	-2.042	-0.355
raw material	2.561	0.791	3.238	0.001	1.116	4.253
plat. depth:thick. eve	1.484	0.522	2.844	0.004	0.536	2.620

(1) not shown because of not having any meaningful interpretation

(2) all covariates were z-transformed to a mean = 0 and sd = 1, original means (sd) were: curvature: 175.13(10.76); epa: 67.26(15.02); thickness evenness: 2.53 (2.18); platform depth: 9.67(6.68)

(3) square root transformed prior to z-transformation

(4) standardized by the geomean

### Discriminant function analysis: predicting archaeological flake group association

**Fig 7:**
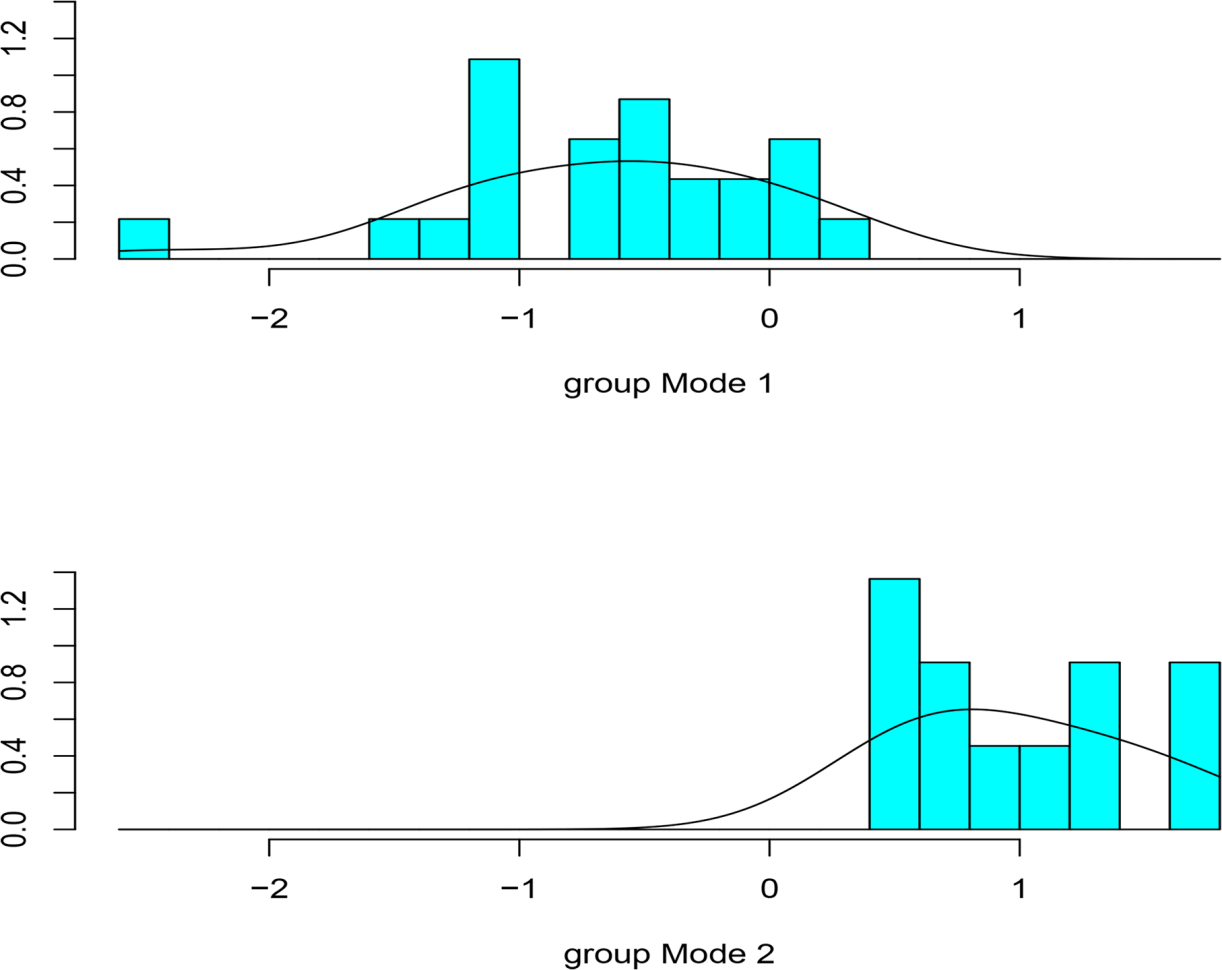
The classification of archaeological material. Histogram showing the results of classifying Elandsfontein Cutting 10 using the discriminant function analysis model built with the experimental dataset.

The whole flakes from Elandsfontein Cutting 10 were classified into Mode 1 (68%) and Mode2 (32%) using the experimental linear discriminant model in its predictive capacity (Fig 7). The posterior probabilities measure the strength of association of each specimen to a specific technological group membership. If the posterior probability is closer to 1 it means that a specimen was confidently assigned to either Mode 1 or Mode 2 group [108]. In our study the majority of archaeological flakes had posterior probabilities that were between 0.8 and 0.9 and the mean of posterior probabilities for the archaeological flakes was equal to 0.8 (sd = 0.2). This suggests that most archaeological flakes were confidently assigned to a given group. Ten out of eleven archaeological flakes classified as Mode 2 were made on silcrete. Only one of the quartz flakes was classified as Mode 2. The majority of the Elandsfontein Cutting 10 flakes (79%) used in our analysis are made on silcrete. Interestingly this contrasts with the high frequency of LCTs made on an igneous rock.

## Discussion and Conclusion

### Influential variables (test predictors) and their association with shaping

The objective of this paper was to quantify and explore the key morphological variables that distinguish between flakes produced during Mode 1 and Mode 2 core reduction. The results of both the discriminant function analysis and Generalized Linear Model demonstrate that curvature and thickness evenness are the most important factors in determining the technological attribution of the archaeological and experimental flakes.

**Fig 8:**
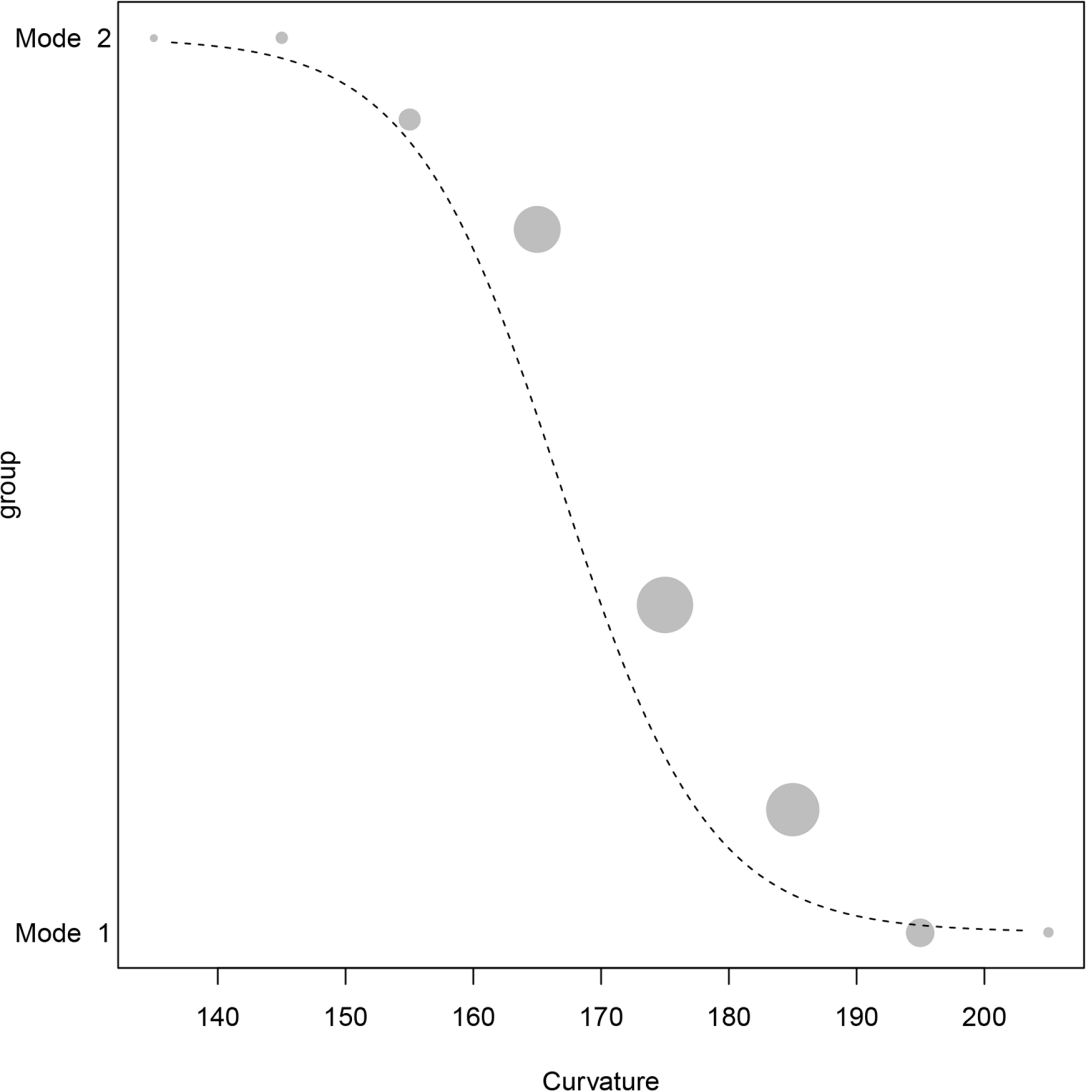
Generalized Linear Model-Curvature. The graph depicts the group association probability as a function of curvature. The dashed line is the fitted model. For visual clarity, curvature was binned in 10 degree sections. The area of the circles corresponds to the sample size per cell. The binned curvature values—which have different areas based on the sample represented—are concentrated between 160 and 170 degrees.

Fig 8 plots group association as a logistic function of curvature at the average values of all other variables (which, as described previously were z-transformed). The plot demonstrates that the Generalized Linear Model fits the experimentally derived data exceptionally well. As the curvature variable is associated with an angle, low values represent flakes that are very curved. Low values for the curvature variable are associated with the Mode 2 group and high values are associated with the Mode 1 group. The inflection point lies between 160 and 180 degrees (Fig 8).

The relative effect of the curvature variable is associated with marked differences in the morphology of the core surfaces from which the flakes were removed. Plano-convex and bi-convex bifacial cores [24] are strikingly different from the cross-sectional shape of the Mode 1 cores. These differences are associated with whole flake profiles from bifacial tool production that are more curved (i.e. low angles in the present study) and less curved Mode 1 flakes (angles larger than 160 in the present study).

**Fig 9:**
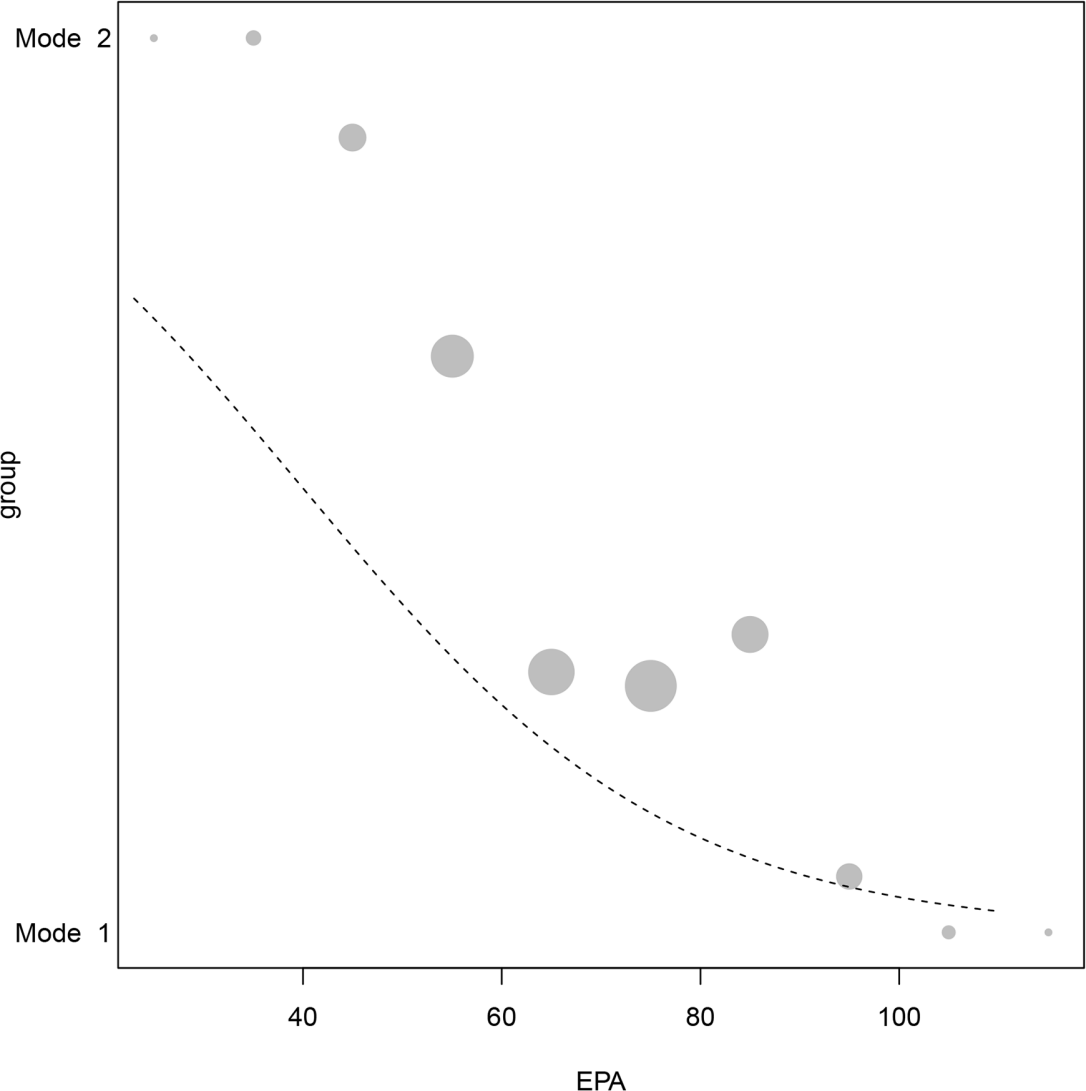
Generalized Linear Model-EPA. The graph depicts the group association probability as a function of EPA. The dashed line is the fitted model. For visual clarity, curvature was binned into 10 degree sections. The area of the circles corresponds to the sample size.

In contrast, although EPA was a statistically significant predictor in the Generalized Linear Model, it has a notably smaller effect on the response (Tables 4 and 6). Fig 9 shows a model based on EPA at the average values of all other predictors. The fitted model does not explain the error distribution in the EPA data well, particularly where values of Mode 1 flakes are displayed on the graph. This result might be explained by the fact that EPA is influential in differentiating Mode 1 and 2 techniques only while interacting with platform depth. However, the interaction between EPA and platform depth was not significant as the comparison between the full model (with the interaction) and the reduced model (without interaction) revealed: χ ^2^ = 239, df = 1, P> 0.05.

Thickness evenness and platform depth played an important role in the discriminant function analysis and Generalized Linear Models as individual predictors (Tables 4 and 6). However, most importantly the interaction tested in the Generalized Linear Model between thickness evenness and platform depth proved to be significant. Fig 6 is a three-dimensional plot depicting platform depth, thickness evenness and group membership on y, x and z axes respectively. Overall, there is a good fit between the data and the model.

The plot reflects the interaction between thickness evenness and platform depth, which is critical in separating technological groups. For flakes with low thickness-evenness values–‘even’ flakes in terms of how thickness is distributed along the length axes—platform size is a relatively good predictor of technological group membership. However as flakes become progressively more uneven and thickness-evenness values increase, platform size is far less decisive—to not being influential at all—in determining whether flakes are associated with Mode 1 or Mode 2 groups. This means, for example, that the predictive strength of the platform depth variable for a given flake is dependent on the values of the thickness evenness variable for that flake.

The significance of the described interaction between thickness evenness and platform depth has implications for the identification of bifacial shaping in the archaeological record. Identifying flakes resulting from bifacial shaping is difficult when looking only at the main *effects* of *individual* morphological variables. Understanding the combined effects of some of these variables is critical in this endeavor. Combined effects–or interactions–make distinguishing bifacial products from flake and core technologies highly complex. The Generalized Linear Model was therefore critical here in that it enabled us to investigate how different aspects of flake morphology behave relative to one another within each technological group. Further, these relationships influence the situations in which certain variables can be accurately used to identify flake production strategies.

### Application to the archaeological materials

Singer & Wymer [34] and later Deacon [33] suggested that LCTs at Elandsfontein Cutting 10 were probably not made on site. Further, they proposed that flakes recovered at this locality were unlikely to be related to the production of LCTs. These suggestions were made based on typological and descriptive observations of the assemblage.

Our quantitative study supports this finding by demonstrating that the majority of flakes in the assemblage are the products of Mode 1 like technological strategies. Our findings confirm the suggestion that the LCTs were indeed made elsewhere and transported to the Elandsfontein Cutting 10 locality in their reduced form. Even though the majority of the flake collection are products of Mode 1 strategies, the proportion of Mode 1 to Mode 2 cores in the collection is slightly smaller (49% are Mode 1 cores, 51% Mode 2 bifacial pieces).

Moreover, 62.5% of the LCTs from Cutting 10 were made on quartz porphyry (27% made on silcrete, and 10.5% made from quartz) while 91% of those archaeological flakes assigned by the discriminant function analysis to Mode 2 were made on silcrete. The absence of quartz porphyry flakes also suggests that LCTs made on this raw material were manufactured and maintained away from the site. In contrast, based on (1) the absence of LCT blanks or preforms, and (2) the presence of Mode 2 silcrete flakes we propose that silcrete LCTs were manufactured away from Cutting 10, but underwent some maintenance at the Cutting 10 locality.

Overall, our analysis indicates the presence of two distinct technological strategies practiced at the Cutting 10 locality. One strategy entails the onsite reduction and discard of Mode 1 cores along with their associated *debitage*. The second strategy is represented by LCT forms, which were transported to the site in a reduced state and then discarded. This suggests that hominins at Elandsfontein Cutting 10 varied decisions regarding the discard and maintenance of the products of different technological systems based on different contextual factors. Current analyses are ongoing in the reconstruction of these contextual factors [117].

Reviews of the time period when the Acheulean first appeared often suggest that Mode 2 technology rapidly replaced Mode 1 technology shortly after the appearance of the Acheulean in the Early Pleistocene [38,73,118]. However, the Elandsfontein Cutting 10 example indicates the importance of Mode 1 cores even in assemblages where the counts of LCTs and cores suggest that LCT production is the major component of the technological strategy. This further supports the assertion that cores/LCTs and detached pieces may reflect subtly different components of a technological system, and perhaps underpin different aspects of hominin tool transport behavior [119]. This suggests that complex and variable behavioral factors may be elucidated by distinguishing Early Stone Age technological systems based on the characteristics of both flake and core assemblage components, and the discussion of quantitatively determined frequencies in this regard.

The rare agreement between this study and previous–methodologically different—independent avenues of investigation [33,34] further validates the results of our analyses. Additionally this agreement suggests that the methodology we present here may be useful in the investigation of other Pleistocene assemblages where traditionally characteristic cores are absent.

## Supporting Information

S1 Table Raw material percentages. Raw materials used in experimental and archaeological assemblages.
(DOCX)

S2 Table Archaeological assemblage. (XLSX)

S3 Table Experimental assemblage. (XLSX)

S1 Text Supporting text: Measurements of the curvature variable. (DOCX)
