# Supplementary material for: Documenting Differences between Early Stone Age Flake Production Systems: An Experimental Model and Archaeological Verification
Source: PLoS One. 2015 Jun 25;10(6):e0130732. doi: 10.1371/journal.pone.0130732 (PMC4482428; doi:10.1371/journal.pone.0130732)
Supplement: S1 Table — Raw materials used in experimental and archaeological assemblages. (DOCX) [file pone.0130732.s001.docx]

**S1 Table. Raw material percentages.**

| Experimental flakes: | % |
| --- | --- |
| silcrete | 68.8 |
| quartzite | 31.2 |
| Archaeological: Cutting 10 | % |
| silcrete | 79.4 |
| quartzite | 5.9 |
| quartz | 5.9 |
| quartz porphyry | 2.9 |
| hornfels | 2.9 |
| sandstone | 2.9 |
