# Supplementary material for: Documenting Differences between Early Stone Age Flake Production Systems: An Experimental Model and Archaeological Verification
Source: PLoS One. 2015 Jun 25;10(6):e0130732. doi: 10.1371/journal.pone.0130732 (PMC4482428; doi:10.1371/journal.pone.0130732)
Supplement: S1 Text — (DOCX) [file pone.0130732.s004.docx]

**S1 Text: Supporting text: Measurements of the curvature variable**

Here we provide information on how we conducted measurements of the curvature variable. To capture the curvature we used Nikon D90 camera. We stabilized flakes in salt so that the flake profiles were visible. To capture curvature, we fixed the flake so that its profile was half way inserted in salt at the point of percussion and then following the technological length axis. The measurements were taken using Image J software and calculations were made in Microsoft Excel.

1. First, we measured the technological length of a flake (L) and then at the half-length (M) we took a perpendicular measurement to the ventral surface of the flake (H).
2. M and H became *catheti* of a right triangle allowing us to find a hypotenuse (С).

С= √(H^2^)+(M^2^)

1. Then we found the inverse cosine between the M and the C.

Cos ^-1^(M/C)

1. We turned the inverse cosine into degrees to get the angle (b).
2. Finally, we found the third angle (a) of the triangle and multiplied it by 2 in order to get the angle for the whole flake.

a=(90-b)*2

1. If the ventral face of the profile was convex with a pronounced bulb as it was often the case with Mode 1 flakes at the end we used “an if” statement in Excel.

If(“convex” then a=360-a)
